# Receptor tyrosine kinases and schistosome reproduction: new targets for chemotherapy

**DOI:** 10.3389/fgene.2014.00238

**Published:** 2014-07-18

**Authors:** Marion Morel, Mathieu Vanderstraete, Steffen Hahnel, Christoph G. Grevelding, Colette Dissous

**Affiliations:** ^1^Center for Infection and Immunity of Lille, INSERM U1019, CNRS-UMR 8204, Institut Pasteur de Lille, University Lille Nord de FranceLille Cedex, France; ^2^Biomedical Centre for Research Seltersberg, Institute of Parasitology, Justus-Liebig-University GiessenGiessen, Germany

**Keywords:** schistosome, receptor tyrosine kinase, signaling, reproduction, chemotherapy

## Abstract

Schistosome parasites still represent a serious public health concern and a major economic problem in developing countries. Pathology of schistosomiasis is mainly due to massive egg production by these parasites and to inflammatory responses raised against the eggs which are trapped in host tissues. Tyrosine kinases (TKs) are key molecules that control cell differentiation and proliferation and they already represent important targets in cancer therapy. During recent years, it has been shown that receptor tyrosine kinases (RTK) signaling was active in reproductive organs and that it could regulate sexual maturation of schistosomes and egg production. This opens interesting perspectives for the control of transmission and pathogenesis of schistosomiasis based on new therapies targeting schistosome RTKs. This review relates the numerous data showing the major roles of kinase signaling in schistosome reproduction. It describes the conserved and particular features of schistosome RTKs, their implication in gametogenesis and reproduction processes and summarizes recent works indicating that RTKs and their signaling partners are interesting chemotherapeutical targets in new programs of control.

## SCHISTOSOMES, KINASE SIGNALING, AND REPRODUCTION

Schistosomiasis, or bilharzia, is a parasitic disease with outstanding medical and economic importance, caused by trematodes of the *Schistosoma* genus (Chitsulo et al., 2004). *Schistosoma mansoni*, *S. haematobium*, and *S. japonicum* are the three major infective species in humans. Whereas most of the trematodes are hermaphrodites, schistosomes have separate sexes, and the sexual maturation of female worms is dependent on constant pairing with males (Popiel and Basch, 1984; LoVerde and Chen, 1991; Kunz, 2001). Pairing induces mitosis and maintains differentiation of stem cell-like precursor cells in the ovary and vitellarium (Erasmus, 1973; Den Hollander and Erasmus, 1985; Kunz, 2001; Galanti et al., 2012). These processes are a prerequisite for the production of eggs, formed by one oocyte and 30–40 vitelline cells combined within the ootype to form viable eggs. The control of this major parasitic disease currently relies on mass treatment with a single drug, Praziquantel. This drug is efficient against the three human schistosome species, but its widespread use raises fears about resistance and motivates the search for alternative therapies (Doenhoff et al., 2008; Melman et al., 2009). During recent years, much effort has been made to understand the development of female reproductive organs with the aim to find strategies to reduce transmission and morbidity of schistosomiasis by preventing egg production (LoVerde et al., 2009; Beckmann et al., 2010a). Indeed, eggs are responsible for parasite transmission but also for pathogenesis in humans since they accumulate in host tissues, particularly in liver, and elicit granulomatous inflammatory reactions leading to periportal fibrosis, portal hypertension, and hepatosplenomegaly (Hoffmann et al., 2002).

Evidence has been obtained that TGF-β pathways play a major role in female reproductive development and egg embryogenesis. Essential components of TGF-β pathways (TbRI and II, R-Smad, Co-Smad, FKBP12) have been identified in schistosomes, and their expression in vitelline cells was demonstrated (Davies et al., 1998; Beall et al., 2000; Osman et al., 2001, 2004, 2006; Knobloch et al., 2004). The TGF-β pathway regulates the synthesis of SmGCP, a gynecophoral canal protein involved in promoting contact between males and females (Osman et al., 2006). The activin ligand of the receptor TbRII, SmInAct, is also crucial for successful embryogenesis in schistosome eggs (Freitas et al., 2007) and the TbRI serine/threonine kinase inhibitor (TRIKI) was shown to reduce vitelline cell mitotic activity and egg production in female worms (Knobloch et al., 2007). Tyrosine kinases (TKs) are also important for female gonad development and in this context, the Src-like cytosolic TK (CTK), SmTK3, seems to play a dominant role in proliferation of vitelline cells (Kapp et al., 2004). Treatment of parasites with the Src inhibitor Herbimycin A significantly blocked male-induced mitotic activity in paired females and interestingly, the combined treatment of paired schistosomes with TRIKI and Herbimycin A was more efficient to reduce mitosis and egg production, indicating possible cross-talk between Src and TGF-β pathways (Knobloch et al., 2007). Recently, transcriptome analyses of inhibitor-treated schistosomes provided further evidence for a cooperation between Src-kinase and TGF-β pathway in the control of mitosis and eggshell formation (Buro et al., 2013) and for an association of Abl-kinase activities with TGFβ signaling (Buro et al., 2014). Finally, the Syk kinase SmTK4 (Knobloch et al., 2002a,b) and the Src/Abl hybrid kinase SmTK6 (Beckmann et al., 2011) presumably act together with SmTK3 in a multi-kinase complex to transduce signals potentially induced by the activation of membrane receptors, such as integrins (Smβ-Int1) or receptor tyrosine kinases (RTKs) and which are important for gametogenesis in parasite gonads (Beckmann et al., 2011, 2012).

## SCHISTOSOME RECEPTOR TYROSINE KINASES

Receptor tyrosine kinasess play essential roles in embryonic development and in various adult tissues and organs, in which they control fundamental processes, such as cell proliferation and differentiation, cell cycle and survival, migration, and metabolism (Ullrich and Schlessinger, 1990). RTKs form a superfamily of transmembrane proteins present in all metazoans, from sponges to humans (Suga et al., 2012) and they are composed of an extracellular ligand binding region formed by various subdomains, a single transmembrane domain, and an intracellular domain with intrinsic TK activity. Annotation of the *S. mansoni* genome (Berriman et al., 2009; Protasio et al., 2012) and analyses by combined computational approaches have shown that the tyrosine kinome of *S. mansoni* contains 15 RTKs including four members of the epidermal growth factor receptor (EGF-R) family, two of the IR (insulin receptor) family, two FGF-R (fibroblast growth factor receptor) members, one representative of Ephrin-R, ROR, and MuSK families, a homolog of CCK4, and one unknown receptor (Andrade et al., 2011; Avelar et al., 2011). Additionally, the schistosome genome encodes two Venus kinase receptors (VKRs), belonging to a novel family of RTKs originally discovered in *S. mansoni* (Vicogne et al., 2003; Dissous et al., 2014a,b).

Over the past few years, five RTKs of *S. mansoni* have been particularly well investigated. SER (*Schistosoma* EGF-R) was the first RTK described in* S. mansoni* (Shoemaker et al., 1992). It contains a conserved intracellular TK domain and an extracellular domain for binding of EGF ligands. When expressed in mammalian cells, SER can bind human EGF with the same affinity as human EGF-R (HER) and it activates the classical and conserved Ras/ERK signaling pathway. Human EGF was shown to induce SER autophosphorylation in *S. mansoni* adult worms and to increase protein and DNA synthesis as well as protein phosphorylation in schistosome larvae, indicating for the first time that host hormones were involved in regulating schistosome development (Vicogne et al., 2004). Such a potential dialog between host ligands and parasite receptors was further confirmed by the ability of human TGF-β to activate TbRI/II schistosome receptors (Beall and Pearce, 2001) but also by evidence that human insulin can bind to schistosome membrane RTKs to regulate metabolic and glucose uptake activities in the parasite (Khayath et al., 2007; Ahier et al., 2008; You et al., 2010). Two members of the IR family were identified in *S. mansoni* (SmIR1 and SmIR2; Khayath et al., 2007), then in *S. japonicum* (SjIR1 and SjIR2; You et al., 2010). Schistosome IR1 and IR2 display differences in the structural motifs essential for signaling and in their expression sites. Schistosome IR1 is expressed in muscles, intestinal epithelial cells and at the basal membrane of the tegument (Khayath et al., 2007), and they are colocalized with SGTP1 and SGTP4, the schistosome glucose transporters involved in glucose uptake (Skelly et al., 1994). Schistosome IR2 is massively expressed in parenchymal cells of adult schistosomes (Khayath et al., 2007; You et al., 2010), and SjIR2 was also localized in vitelline cells (You et al., 2010). A single IR is present in most invertebrate species which regulates both metabolism and growth, while two receptors IR and IGF-1R exist in vertebrates, which are specialized in metabolic and glucose uptake regulation, and in growth control, respectively (Kim and Accili, 2002). It was suggested that similarly SmIR1 and SjIR1 could be specialized in sugar uptake and SmIR2 and SjIR2 preferentially involved in growth of schistosomes.

Besides these conventional and conserved RTKs, which are able to respond to host growth factors (EGF, insulin) and to activate conserved signaling pathways, schistosomes were shown to express unconventional RTKs, named VKRs for Venus kinase receptors (Vicogne et al., 2003; Ahier et al., 2009). VKRs are composed of an extracellular Venus flytrap module (VFT), linked through a single transmembrane fragment to an intracellular TK domain similar to that of IRs (Vicogne et al., 2003). VFTs are the extracellular domains of many G-protein coupled receptors of class C, and they are composed of two lobes that close upon the binding of small ligands (amino-acids, ions) similarly to the leaves of the Venus flytrap carnivorous plant, *Dionaea muscipula*, when it catches its prey (Pin et al., 2003). VKRs are found in a large variety of invertebrates from cnidarians to echinoderms (Ahier et al., 2009; Vanderstraete et al., 2013a), and are highly expressed in larval stages and in gonads, suggesting a role of these proteins in embryonic and larval development as well as in reproduction (Ahier et al., 2009; Vanderstraete et al., 2014). *Vkr* genes are found as single copies in most species but in platyhelminths two different *vkr* copies are present (Gouignard et al., 2012; Vanderstraete et al., 2013a, 2014). Up to now, two VKRs have been found in *S. mansoni* as well as in *S. haematobium* (Young et al., 2012). SmVKR1 and SmVKR2 of *S. mansoni* have been extensively studied. They were shown to be activated by L-arginine and calcium ions, respectively (Gouignard et al., 2012) and, as many other RTKs, VKRs dimerize to induce intracellular pathways involved in protein synthesis and cellular growth, like the MAPK and PI3K/Akt/S6K pathways (Dissous et al., 2014a; Vanderstraete et al., 2014).

**FIGURE 1:**
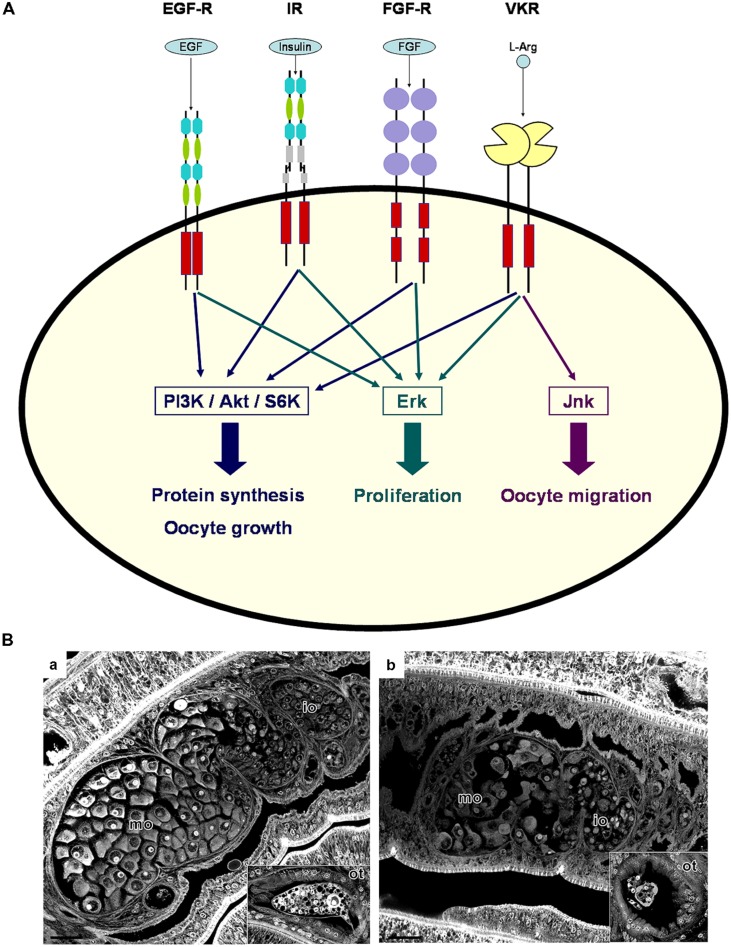
**Receptor tyrosine kinase (RTK) signaling in schistosome gonads. (A)** Following activation by their respective ligands, schistosome RTKs (EGF-R, IR, FGF-R, and VKR) expressed in gonads can induce protein synthesis, cell growth and proliferation by activating the PI3K/Akt/S6K and Erk MAPK pathways. VKR activation was shown to also activate the Jnk MAPK pathway, potentially involved in oocyte migration. **(B)** Morphology of the reproductive organs of *S. mansoni* females is affected by *dsSmvkr* RNA interference. Worms were treated with control irrelevant **(A)** or *dsSmvkr* **(B)** dsRNAs (as described in Vanderstraete et al., 2014). Whole-mount preparations were stained with carmine red and examined by confocal laser scanning microscopy. In control worms (a), we observe immature oocytes (io) within the smaller, anterior part of the ovary and big and mature oocytes (mo) in the posterior part. The ootype (ot) contains a viable egg formed by one oocyte and vitelline cells. Treatment with *dsSmvkr* (b) induced a strong disorganization and size reduction of the ovary, as well as the abortion of egg formation. Scale bar: 20 μm.

Among the growth factor receptor panel, EGFR and IR/IGFR have been shown to play essential roles in mammals (Schneider and Wolf, 2008; Sirotkin, 2011) as well as in insects (Graf et al., 1997; Brown et al., 2008; Parrott et al., 2012) for the control of ovarian functions and reproductive processes. Additionally, the discovery that VKRs were abundantly transcribed in the gonads of many invertebrate species (Ahier et al., 2009), indicated that VKR signaling could also participate in reproductive activities. Further analyses have confirmed that all these RTKs were potentially involved in reproduction processes in schistosomes (**Figure 1A**).

## FUNCTIONS OF SCHISTOSOME RTKs IN REPRODUCTION

Besides the importance of the receptor Ser/Thr (S/T) kinases SmTbRI/II and of TGF-β-dependent pathways in schistosome reproduction processes (LoVerde et al., 2007, 2009), TK signaling is also predominant for schistosome gonad development (Beckmann et al., 2010a,b), and a possible cooperation between STK and TK pathways has been proposed for the control of mitosis and eggshell formation in these parasites (Knobloch et al., 2006; Buro et al., 2013). Moreover, diverse CTKs of *S. mansoni* (SmTK3, SmTK4, and SmTK6) were shown to form complexes susceptible to participate in RTK signaling in gonads (Beckmann et al., 2011).

The EGF receptor SER is transcribed in the vitellarium and ovary of schistosome females together with its potential substrate SmEps8 (EGF-R kinase substrate 8) and SmTK3, the CTK that was shown to play a dominant role in proliferation of vitelline cells (Kapp et al., 2004). Since SmEps8 interacts in yeast two-hybrid (Y2H) assays with SmTK3, this might indicate a possible role of SER and EGF pathways in reproductive activities (Beckmann et al., 2010a).

Insulin pathways, and especially the insulin-mediated PI3K pathway, play major roles in reproduction processes in many organisms (Graf et al., 1997; Brown et al., 2008). Moreover, the TOR (target of rapamycin)/S6K (p70 S6 kinase) pathway has been identified as an essential nutrient-sensing tool regulating egg development under the control of insulin in mosquitoes (Arsic and Guerin, 2008). Limited information has been obtained concerning the importance of IR receptors in the development of schistosome gonads, except that vaccination of mice with the purified insulin-binding domain of SjIR2 provoked a retardation of adult *S. japonicum* growth and a substantial decrease of egg maturation and laying in parasitized animals (You et al., 2012). While transcriptome analyses did not highlight an overexpression of IR transcripts neither in *S. mansoni* (Nawaratna et al., 2011) nor in *S. japonicum* (Gobert et al., 2009) gonads, they indicated an overexpression of VKR-like transcripts in ovary and vitelline cells of *S. japonicum* (Gobert et al., 2009). In *S. mansoni*, quantitative-PCR results confirmed that both *Smvkr1* and *Smvkr2* genes were more actively transcribed in female worms than in males (Gouignard et al., 2012). By *in situ* hybridization, *Smvkr1* and *Smvkr2* transcripts were detected in testes but they were more abundant in ovaries, in which the expression profile of each *vkr* was noticeably different. *Smvkr1* transcripts were present mainly in the posterior part of the ovary that contains mature oocytes (in prophase I of meiosis) whereas *Smvkr2* transcripts were found in the anterior part of the organ containing immature oocytes. Q-PCR data obtained from isolated ovaries (Hahnel et al., 2013) indicated that both *Smvkr1* and *Smvkr2* transcripts were up-regulated strongly in the ovaries of sexually developed females as compared to the organs from virgin females issued from unisexual infections. Additionally, two RTKs of the FGFR family, SmFGFR-A and SmFGFR-B were found among the genes identified as up-regulated in female gonads after pairing (Hahnel et al., 2014, this issue). Analyses of isolated testes confirmed the presence of *Smvkr1* and *Smvkr2* transcripts in male reproductive organs and showed similarly their up-regulation in testes from males issued from bisexual infections (Vanderstraete et al., 2014). This indicated the particular importance of SmVKR receptors during the development and maturation of schistosome reproductive organs.

Recently, molecular partners of SmVKRs have been identified from Y2H screening of an adult *S. mansoni* cDNA library with active intracellular domains of VKRs as baits. The analyses of the resulting partners indicated similarities between VKR and IR pathways, which is in agreement with the identities already observed within the intracellular domains of these RTKs. All the assumptions made from the nature of these partners about the specificity of phospho-pathways elicited by VKR, were supported by further studies of VKR activation and signaling in *Xenopus* oocytes. It was shown that ligand-activated VKRs induced, similarly to the endogenous insulin-activated Xenopus IR, the phosphorylation of Erk1/2, Akt, and p70S6K (Vanderstraete et al., 2014). This indicated that VKRs were able to stimulate protein synthesis and cellular growth, as IRs do. The JNK pathway was activated by SmVKR1 but not by SmVKR2, corroborating Y2H screening results which showed a specific interaction of SmVKR1 with Rho1, Mek7, and PP2C (Vanderstraete et al., 2014). Since the JNK pathway has been shown to play a major role in oogenesis and meiosis resumption in *Caenorhabditis elegans* (Smith et al., 2002), in *Drosophila melanogaster* (Sackton et al., 2007), and in mammals (Huang et al., 2011; Chuderland et al., 2012), it was postulated that it could be used as a pathway by SmVKR1 to influence oocyte maturation. Furthermore the implication of schistosome VKRs in oogenesis and spermatogenesis was demonstrated by RNA interference. SmVKR silencing led to an important disorganization of the antero-posterior structure of the ovary and the knock down of *Smvkr1* resulted in the accumulation of big oocytes in the ovary and the absence of egg formation (**Figure 1B**). In male testes, silencing of both *Smvkr* provoked a decrease of cell density within testicular lobes and paucity of sperm, confirming the potential importance of VKRs in reproduction processes (Vanderstraete et al., 2014).

## RTKs AS POTENTIAL TARGETS FOR THE CONTROL OF SCHISTOSOMIASIS

Given the oncogenic role of aberrant signaling from RTKs in humans, these receptors have become attractive therapeutic targets. This led to the generation and the use in therapy of a large number of TK inhibitory compounds. Some of them were named as “tyrphostins” (TYRosine PHOSphorylation INnhibitors; Levitzki and Mishani, 2006), compounds that are able to inhibit multiple RTKs. We have seen that growth factor receptor and TK signaling molecules likely represent key molecules for the development and reproductive activity processes in schistosome worms. Consequently, these molecules are considered as potential targets for novel therapies against schistosomiasis today (Dissous et al., 2007; Dissous and Grevelding, 2011). IR inhibitors [tyrphostins AG1024, AG538, and HNMPA-(AM)3] can potentially affect survival of *S. mansoni* (Ahier et al., 2008) and *S. japonicum* (You et al., 2010) adult worms by blocking the uptake of glucose, an essential nutrient for schistosomes. Imatinib (Gleevec), used in the treatment of multiple cancers but targeting notably the chimeric oncogene BCR-Abl responsible for chronic myelogenous leukemia (Manley et al., 2002) has been described for its fatal impact on morphology, pairing stability, and survival of adult *S. mansoni in vitro* (Beckmann and Grevelding, 2010). Other TK inhibitors have also revealed a potential usefulness for the prevention of egg production by schistosomes. The Src kinase inhibitor, Herbimycin A, was demonstrated to block mitotic activity, expression of eggshell protein gene and egg production in *S. mansoni* female worms, preferentially inactivating the parasite Src-related SmTK3 and causing its degradation (Knobloch et al., 2006). The Syk inhibitor Piceatannol blocked the kinase activity of SmTK4 of *S. mansoni* and reduced egg production by female worms *in vitro* (Beckmann et al., 2010b). Furthermore, the angiokinase inhibitor BIBF1120 was shown to block the activities of the FGFR receptors SmFGFR-A and SmFGFR-B from *S. mansoni* leading to severe effects on the morphology of gonad tissues and the gastrodermis, on reproduction leading to reduced egg production, and finally on worm vitality (Hahnel et al., 2014, this issue).

**FIGURE 2:**
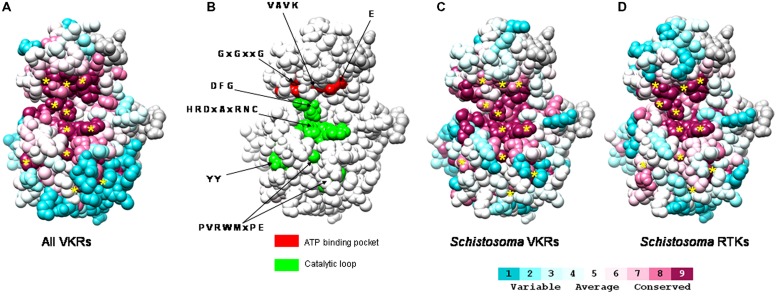
**Conservation of schistosome RTK tyrosine kinase catalytic sites.** An evolutionary trace analysis of the conservation of residues in TK domains was performed using sequence alignment of **(A)** 40 VKRs already known in various invertebrate species (Vanderstraete et al., 2013a), **(C)** four VKRs of *Schistosoma* (SmVKR1 AAL67949.1 and SmVKR2 ADD91576.1, and two VKRs of *Schistosoma haematobium* Sha_103537 and Sha_104501), **(D)** various* Schistosoma* RTKs (EGF, insulin and VKR receptors) including SmVKR1, SmVKR2, the two ShVKRs, SmIR1 (AAN39120), SmIR2 (AAV65745.2), SjIR1 (ACT20714.1), SjIR2 (ACT20715.1), and SER (AAA29866.1). Visualization of the conservation was performed on the human IR TK crystal structure (PDB accession number 1IRK; Ahier et al., 2009). The alignment generated with ClustalW was submitted to the ConSurf website server (http://consurf.tau.ac.il; Landau et al., 2005). Conservation scores of each residue were calculated by taking into account the phylogenetic relationships among the sequences and the similarity between the amino acids in the alignment. Conservation scores are according to a color scale from variable (blue) to conserved (purple) residues. In **(B)**, are indicated the crucial residues of the ATP binding pocket (red) and of the catalytic loop (green) required for kinase activity. The evolutionary trace analyses revealed that these crucial residues (indicated by * in **A,C,D**) are highly conserved among all VKRs **(A,C)** and among the panel of *Schistosoma* RTKs (EGF, insulin, and VKR receptors) from the three human species (*mansoni, haematobium,* and* japonicum*).

More recently, tyrphostin AG1024 emerged as a potent drug molecule that caused dramatic effects both on the viability of larvae of *S. mansoni* and on the fertility of adult worms. The remarkable efficacy of this TK inhibitor (used at μM doses) on parasites was shown to be due to its dual action on the parasite IR and VKR kinases that contain similar catalytic domains (Vanderstraete et al., 2013b). **Figure 2** illustrates the conservation of the catalytic pocket of RTKs characterized in the three human schistosome species. Using a sequence alignment of the TK domain of the various parasite RTKs and the crystal structure of the TK domain of the human insulin receptor, an evolutionary trace analysis revealed that in kinase domains the residues were highly conserved, and particularly those composing the catalytic loop and the ATP-binding site essential for kinase activity (**Figures 2B–D**). These residues were also identical among all the VKRs characterized in other invertebrate species (**Figure 2A**). The conservation of the motifs essential for TK activity in the various schistosome RTKs is very likely accounting for the efficacy of AG1024 to inhibit simultaneously schistosome IR, VKR but also EGFR kinase activities. This was confirmed by the high sensitivity of all these recombinant parasite kinases to AG1024 shown in kinase assays (Dissous et al., 2014b; Vanderstraete et al., 2014). In females of *S. mansoni* treated with AG1024, important size reduction and disorganization of the ovary were observed together with an inhibition of egg production. AG1024 also affected spermatogenesis in males, and all these data confirmed the potential of AG1024 to affect schistosome reproduction by targeting multiple RTKs. The implication of these RTKs in gametogenesis and reproduction processes are valuable reasons to consider them as interesting targets in new control programs, with the main advantage in the case of VKR that their counterparts are absent from the vertebrate host kinase panel. Structural divergences between catalytic domains of host and schistosome RTKs should now be exploited for the design of molecules able to target schistosome RTKs without affecting human host kinases. This should require for the parasite RTK kinase domains the obtention of X-ray crystal structures that would serve as the basis for designing new pharmaceuticals against schistosomiasis. Alternatively, the use of small molecules as antagonist ligands of the VFT domains of schistosome VKRs should be efficient to interfere specifically with VKR pathways and parasite reproduction.

## Conflict of Interest Statement

The authors declare that the research was conducted in the absence of any commercial or financial relationships that could be construed as a potential conflict of interest.
